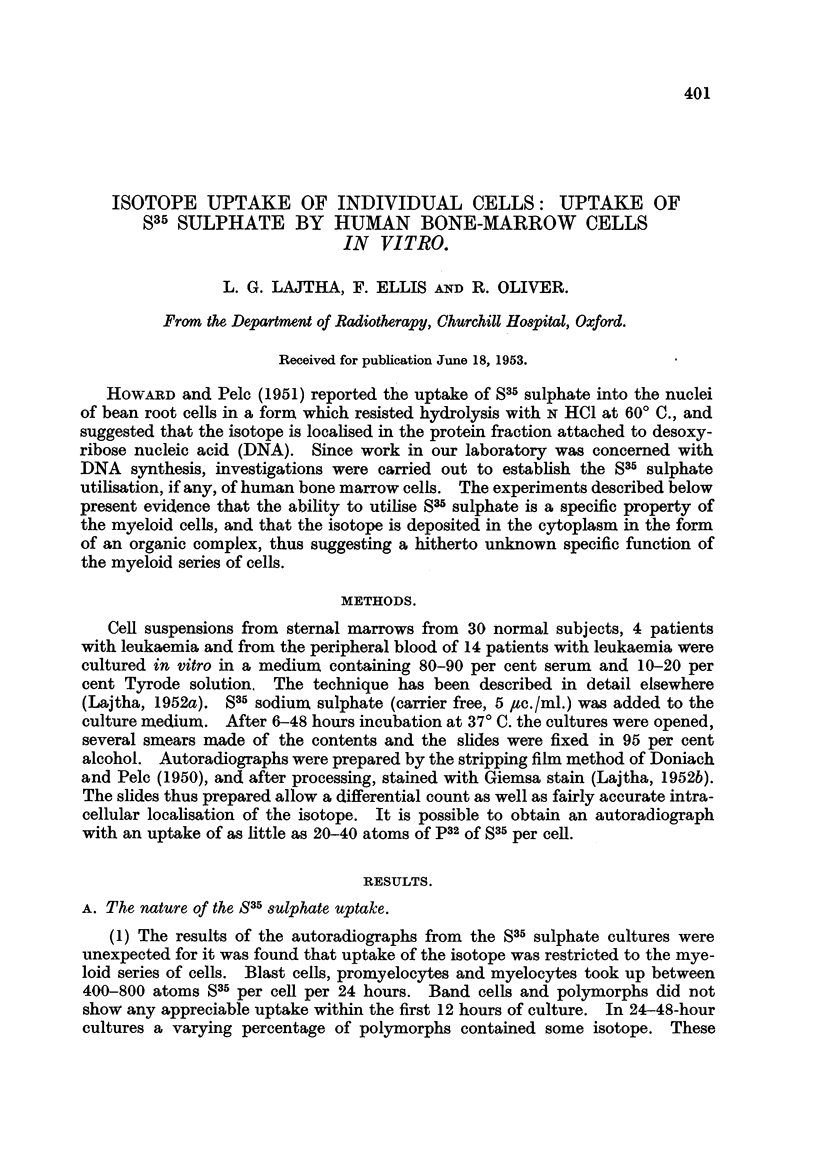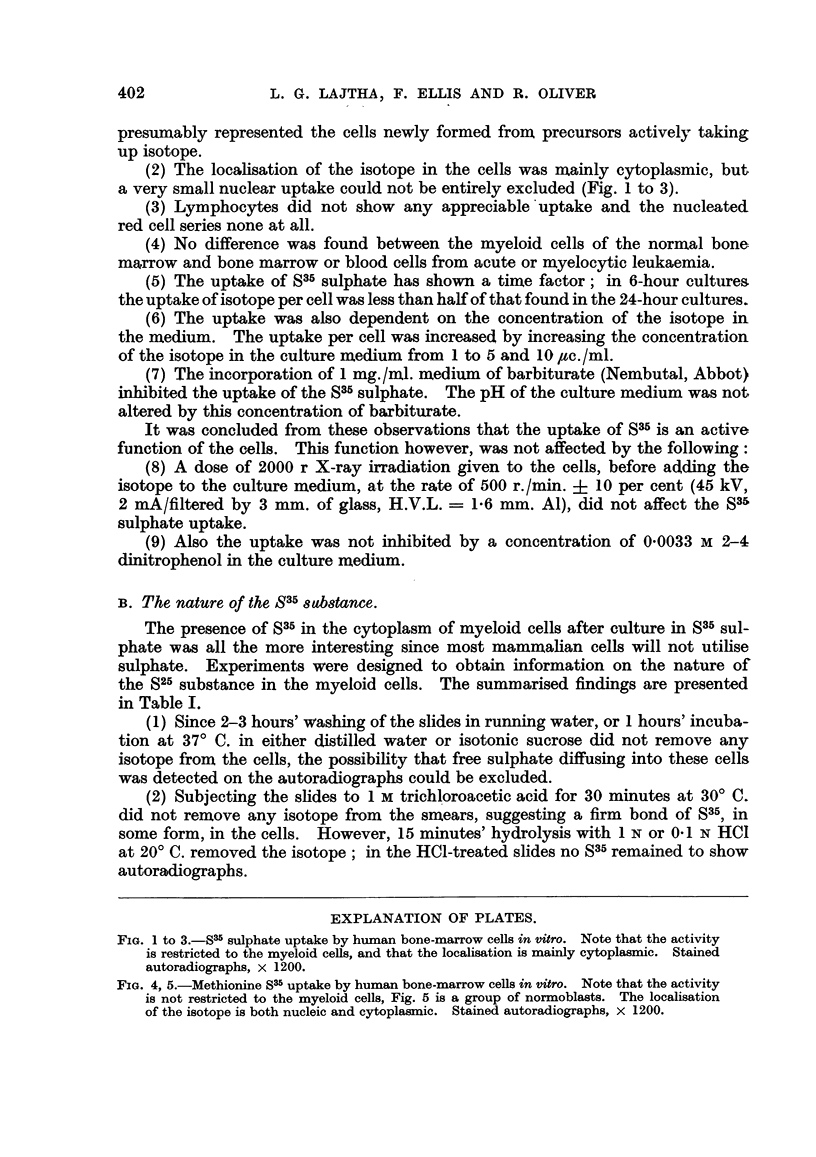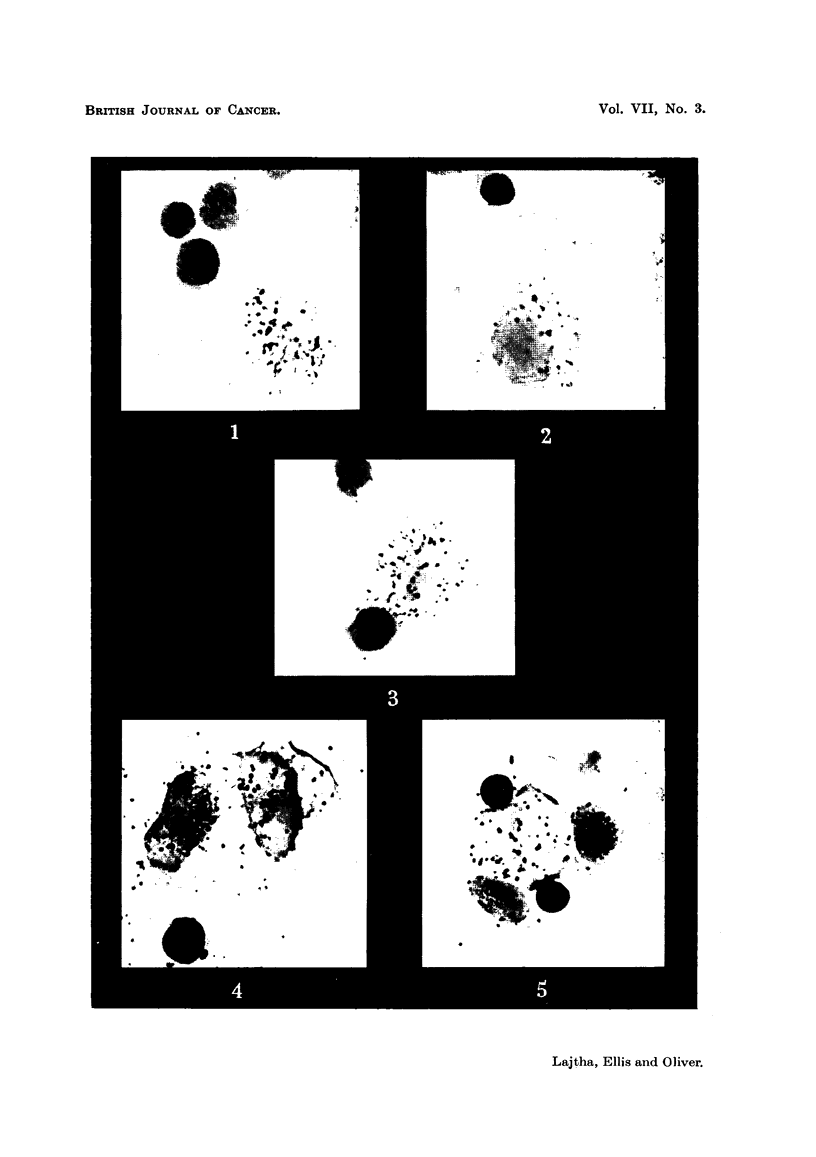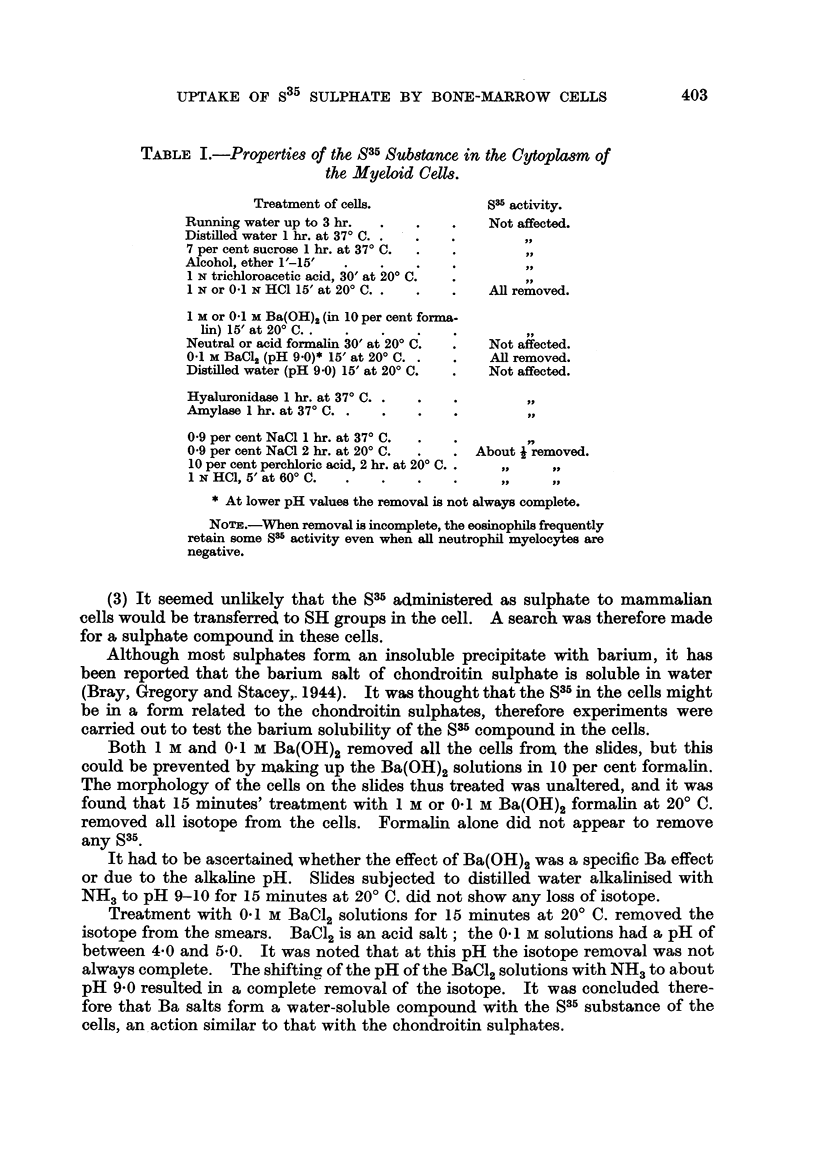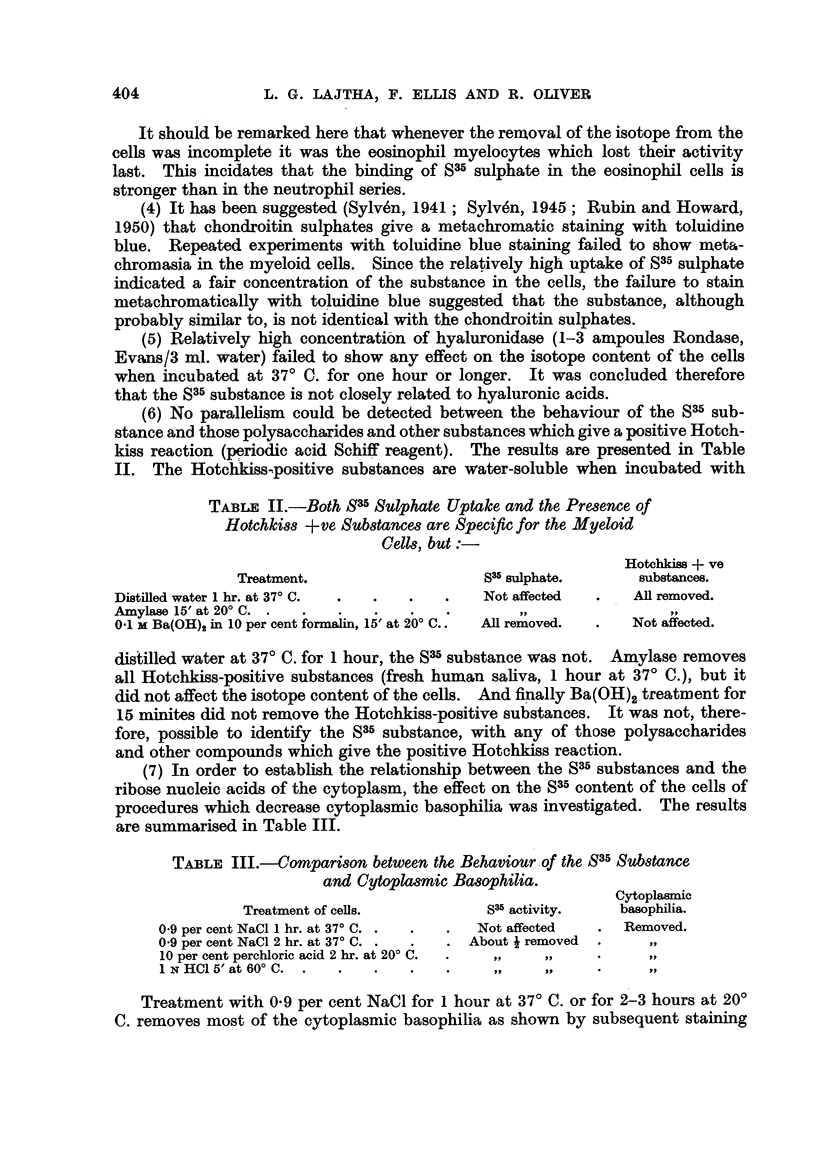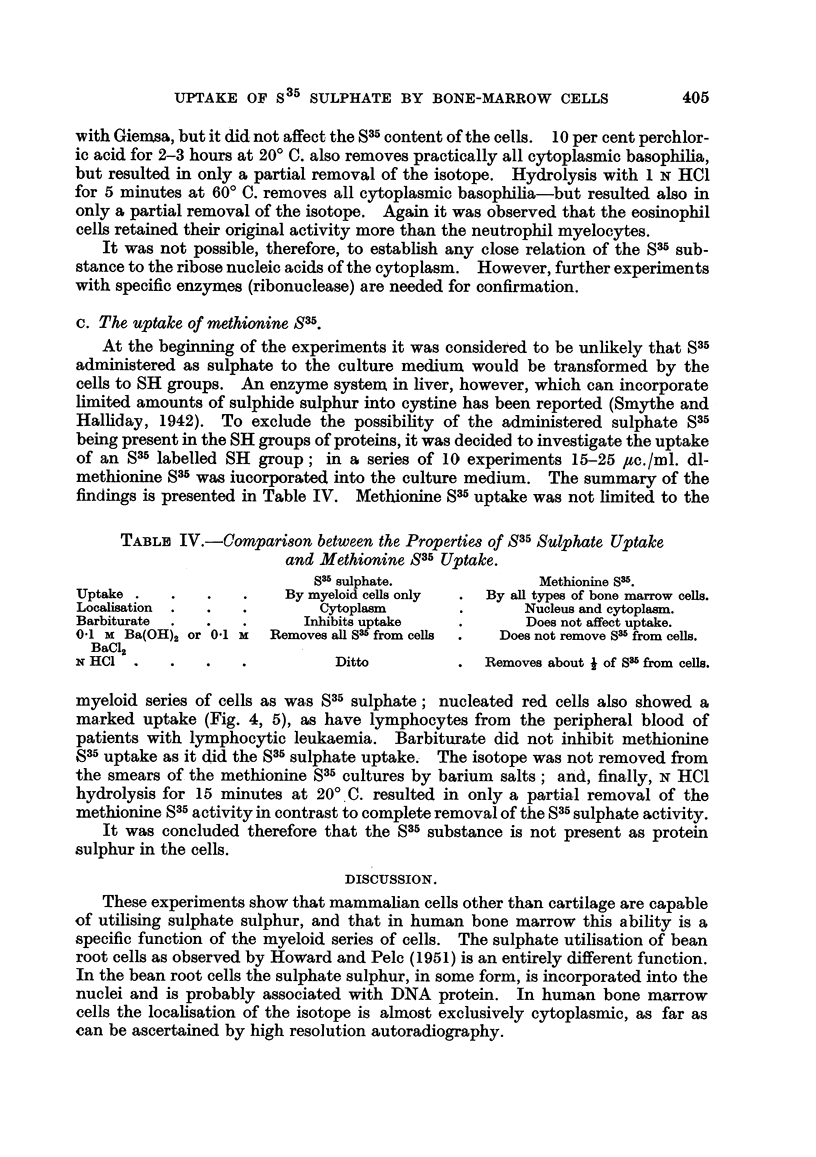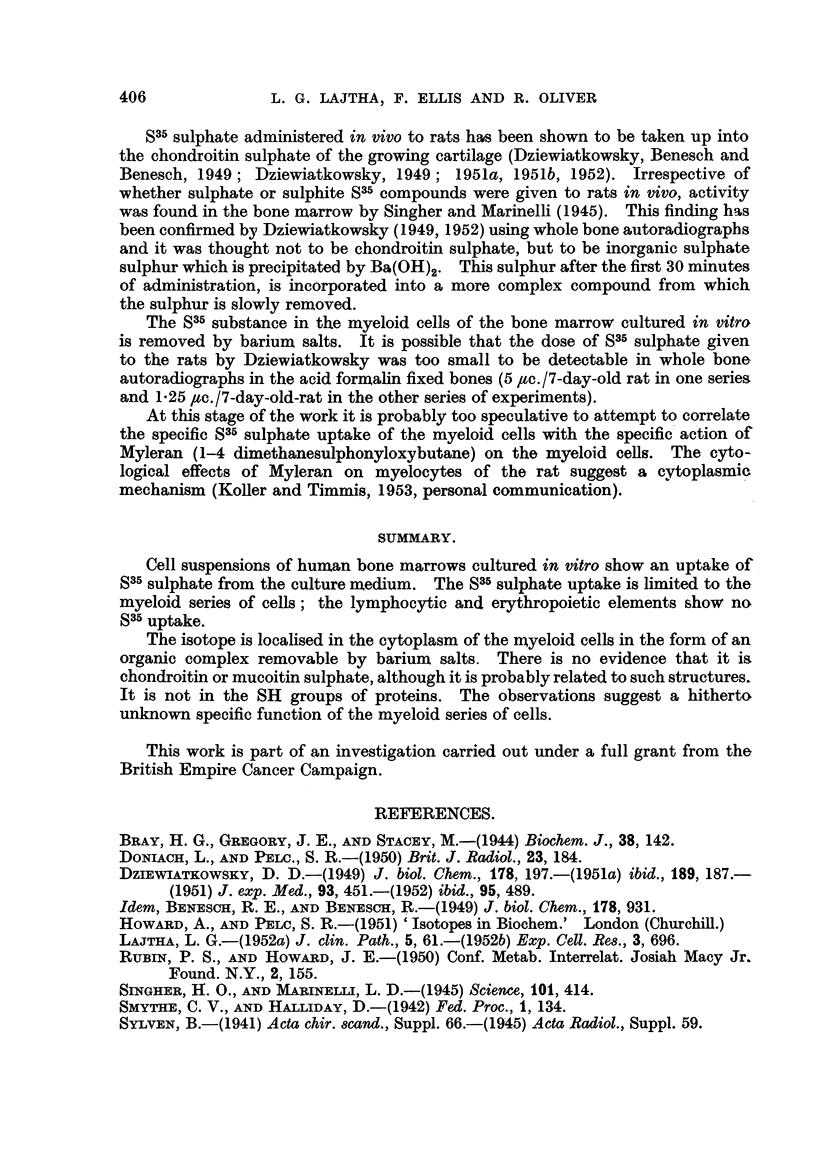# Isotope Uptake of Individual Cells: Uptake of S^35^ Sulphate by Human Bone-Marrow Cells In Vitro

**DOI:** 10.1038/bjc.1953.38

**Published:** 1953-09

**Authors:** L. G. Lajtha, F. Ellis, R. Oliver

## Abstract

**Images:**


### ISOTOPE UPTAKE OF INDIVIDUAL CELLS: UPTAKE OF $S^{35}$ SULPHATE BY HUMAN BONE-MARROW CELLS *IN VITRO.*

L. G. LAJTHA, F. ELLIS AND R. OLIVER.

*From the Department of Radiotherapy, Churchill Hospital, Oxford.*

Received for publication June 18, 1953.

HOWARD and Pelc (1951) reported the uptake of $S^{35}$ sulphate into the nuclei of bean root cells in a form which resisted hydrolysis with N HCl at 60° C., and suggested that the isotope is localised in the protein fraction attached to desoxyribose nucleic acid (DNA). Since work in our laboratory was concerned with DNA synthesis, investigations were carried out to establish the $S^{35}$ sulphate utilisation, if any, of human bone marrow cells. The experiments described below present evidence that the ability to utilise $S^{35}$ sulphate is a specific property of the myeloid cells, and that the isotope is deposited in the cytoplasm in the form of an organic complex, thus suggesting a hitherto unknown specific function of the myeloid series of cells.

### METHODS.

Cell suspensions from sternal marrows from 30 normal subjects, 4 patients with leukaemia and from the peripheral blood of 14 patients with leukaemia were cultured *in vitro* in a medium containing 80–90 per cent serum and 10–20 per cent Tyrode solution. The technique has been described in detail elsewhere (Lajtha, 1952*a*). $S^{35}$ sodium sulphate (carrier free, 5 $\mu$c./ml.) was added to the culture medium. After 6–48 hours incubation at 37° C. the cultures were opened, several smears made of the contents and the slides were fixed in 95 per cent alcohol. Autoradiographs were prepared by the stripping film method of Doniach and Pelc (1950), and after processing, stained with Giemsa stain (Lajtha, 1952*b*). The slides thus prepared allow a differential count as well as fairly accurate intracellular localisation of the isotope. It is possible to obtain an autoradiograph with an uptake of as little as 20–40 atoms of $P^{32}$ of $S^{35}$ per cell.

### RESULTS.

### A. *The nature of the $S^{35}$ sulphate uptake.*

(1) The results of the autoradiographs from the $S^{35}$ sulphate cultures were unexpected for it was found that uptake of the isotope was restricted to the myeloid series of cells. Blast cells, promyelocytes and myelocytes took up between 400–800 atoms $S^{35}$ per cell per 24 hours. Band cells and polymorphs did not show any appreciable uptake within the first 12 hours of culture. In 24–48-hour cultures a varying percentage of polymorphs contained some isotope. These presumably represented the cells newly formed from precursors actively taking up isotope.

(2) The localisation of the isotope in the cells was mainly cytoplasmic, but a very small nuclear uptake could not be entirely excluded (Fig. 1 to 3).

(3) Lymphocytes did not show any appreciable uptake and the nucleated red cell series none at all.

(4) No difference was found between the myeloid cells of the normal bone marrow and bone marrow or blood cells from acute or myelocytic leukaemia.

(5) The uptake of $S^{35}$ sulphate has shown a time factor ; in 6-hour cultures the uptake of isotope per cell was less than half of that found in the 24-hour cultures.

(6) The uptake was also dependent on the concentration of the isotope in the medium. The uptake per cell was increased by increasing the concentration of the isotope in the culture medium from 1 to 5 and 10 $\mu$c./ml.

(7) The incorporation of 1 mg./ml. medium of barbiturate (Nembutal, Abbot) inhibited the uptake of the $S^{35}$ sulphate. The pH of the culture medium was not altered by this concentration of barbiturate.

It was concluded from these observations that the uptake of $S^{35}$ is an active function of the cells. This function however, was not affected by the following :

(8) A dose of 2000 r X-ray irradiation given to the cells, before adding the isotope to the culture medium, at the rate of 500 r./min. $\pm$ 10 per cent (45 kV, 2 mA/filtered by 3 mm. of glass, H.V.L. = 1·6 mm. Al), did not affect the $S^{35}$ sulphate uptake.

(9) Also the uptake was not inhibited by a concentration of 0·0033 M 2–4 dinitrophenol in the culture medium.

B. *The nature of the $S^{35}$ substance.*

The presence of $S^{35}$ in the cytoplasm of myeloid cells after culture in $S^{35}$ sulphate was all the more interesting since most mammalian cells will not utilise sulphate. Experiments were designed to obtain information on the nature of the $S^{25}$ substance in the myeloid cells. The summarised findings are presented in Table I.

(1) Since 2–3 hours' washing of the slides in running water, or 1 hours' incubation at 37° C. in either distilled water or isotonic sucrose did not remove any isotope from the cells, the possibility that free sulphate diffusing into these cells was detected on the autoradiographs could be excluded.

(2) Subjecting the slides to 1 M trichloroacetic acid for 30 minutes at 30° C. did not remove any isotope from the smears, suggesting a firm bond of $S^{35}$, in some form, in the cells. However, 15 minutes' hydrolysis with 1 N or 0·1 N HCl at 20° C. removed the isotope ; in the HCl-treated slides no $S^{35}$ remained to show autoradiographs.

---

EXPLANATION OF PLATES.

FIG. 1 to 3.—$S^{35}$ sulphate uptake by human bone-marrow cells *in vitro*. Note that the activity is restricted to the myeloid cells, and that the localisation is mainly cytoplasmic. Stained autoradiographs, × 1200.

FIG. 4, 5.—Methionine $S^{35}$ uptake by human bone-marrow cells *in vitro*. Note that the activity is not restricted to the myeloid cells, Fig. 5 is a group of normoblasts. The localisation of the isotope is both nucleic and cytoplasmic. Stained autoradiographs, × 1200.

1 2 3 4 5

Lajtha, Ellis and Oliver.

TABLE I.—*Properties of the $S^{35}$ Substance in the Cytoplasm of the Myeloid Cells.*

| Treatment of cells. | $S^{35}$ activity. |
|---|---|
| Running water up to 3 hr. | Not affected. |
| Distilled water 1 hr. at 37° C. | " |
| 7 per cent sucrose 1 hr. at 37° C. | " |
| Alcohol, ether 1′–15′ | " |
| 1 N trichloroacetic acid, 30′ at 20° C. | " |
| 1 N or 0·1 N HCl 15′ at 20° C. | All removed. |
| 1 M or 0·1 M $Ba(OH)_2$ (in 10 per cent formalin) 15′ at 20° C. | " |
| Neutral or acid formalin 30′ at 20° C. | Not affected. |
| 0·1 M $BaCl_2$ (pH 9·0)* 15′ at 20° C. | All removed. |
| Distilled water (pH 9·0) 15′ at 20° C. | Not affected. |
| Hyaluronidase 1 hr. at 37° C. | " |
| Amylase 1 hr. at 37° C. | " |
| 0·9 per cent NaCl 1 hr. at 37° C. | " |
| 0·9 per cent NaCl 2 hr. at 20° C. | About ½ removed. |
| 10 per cent perchloric acid, 2 hr. at 20° C. | " " |
| 1 N HCl, 5′ at 60° C. | " " |

* At lower pH values the removal is not always complete.

NOTE.—When removal is incomplete, the eosinophils frequently retain some $S^{35}$ activity even when all neutrophil myelocytes are negative.

(3) It seemed unlikely that the $S^{35}$ administered as sulphate to mammalian cells would be transferred to SH groups in the cell. A search was therefore made for a sulphate compound in these cells.

Although most sulphates form an insoluble precipitate with barium, it has been reported that the barium salt of chondroitin sulphate is soluble in water (Bray, Gregory and Stacey, 1944). It was thought that the $S^{35}$ in the cells might be in a form related to the chondroitin sulphates, therefore experiments were carried out to test the barium solubility of the $S^{35}$ compound in the cells.

Both 1 M and 0·1 M $Ba(OH)_2$ removed all the cells from the slides, but this could be prevented by making up the $Ba(OH)_2$ solutions in 10 per cent formalin. The morphology of the cells on the slides thus treated was unaltered, and it was found that 15 minutes' treatment with 1 M or 0·1 M $Ba(OH)_2$ formalin at 20° C. removed all isotope from the cells. Formalin alone did not appear to remove any $S^{35}$.

It had to be ascertained whether the effect of $Ba(OH)_2$ was a specific Ba effect or due to the alkaline pH. Slides subjected to distilled water alkalinised with $NH_3$ to pH 9–10 for 15 minutes at 20° C. did not show any loss of isotope.

Treatment with 0·1 M $BaCl_2$ solutions for 15 minutes at 20° C. removed the isotope from the smears. $BaCl_2$ is an acid salt ; the 0·1 M solutions had a pH of between 4·0 and 5·0. It was noted that at this pH the isotope removal was not always complete. The shifting of the pH of the $BaCl_2$ solutions with $NH_3$ to about pH 9·0 resulted in a complete removal of the isotope. It was concluded therefore that Ba salts form a water-soluble compound with the $S^{35}$ substance of the cells, an action similar to that with the chondroitin sulphates.

It should be remarked here that whenever the removal of the isotope from the cells was incomplete it was the eosinophil myelocytes which lost their activity last. This incidates that the binding of $S^{35}$ sulphate in the eosinophil cells is stronger than in the neutrophil series.

(4) It has been suggested (Sylvén, 1941 ; Sylvén, 1945 ; Rubin and Howard, 1950) that chondroitin sulphates give a metachromatic staining with toluidine blue. Repeated experiments with toluidine blue staining failed to show metachromasia in the myeloid cells. Since the relatively high uptake of $S^{35}$ sulphate indicated a fair concentration of the substance in the cells, the failure to stain metachromatically with toluidine blue suggested that the substance, although probably similar to, is not identical with the chondroitin sulphates.

(5) Relatively high concentration of hyaluronidase (1–3 ampoules Rondase, Evans/3 ml. water) failed to show any effect on the isotope content of the cells when incubated at 37° C. for one hour or longer. It was concluded therefore that the $S^{35}$ substance is not closely related to hyaluronic acids.

(6) No parallelism could be detected between the behaviour of the $S^{35}$ substance and those polysaccharides and other substances which give a positive Hotchkiss reaction (periodic acid Schiff reagent). The results are presented in Table II. The Hotchkiss-positive substances are water-soluble when incubated with

TABLE II.—*Both $S^{35}$ Sulphate Uptake and the Presence of Hotchkiss +ve Substances are Specific for the Myeloid Cells, but :—*

| Treatment. | $S^{35}$ sulphate. | Hotchkiss + ve substances. |
|---|---|---|
| Distilled water 1 hr. at 37° C. . . . . . | Not affected . | All removed. |
| Amylase 15′ at 20° C. . . . . . . . | ,, | ,, |
| 0·1 M $Ba(OH)_2$ in 10 per cent formalin, 15′ at 20° C. . | All removed. . | Not affected. |

distilled water at 37° C. for 1 hour, the $S^{35}$ substance was not. Amylase removes all Hotchkiss-positive substances (fresh human saliva, 1 hour at 37° C.), but it did not affect the isotope content of the cells. And finally $Ba(OH)_2$ treatment for 15 minites did not remove the Hotchkiss-positive substances. It was not, therefore, possible to identify the $S^{35}$ substance, with any of those polysaccharides and other compounds which give the positive Hotchkiss reaction.

(7) In order to establish the relationship between the $S^{35}$ substances and the ribose nucleic acids of the cytoplasm, the effect on the $S^{35}$ content of the cells of procedures which decrease cytoplasmic basophilia was investigated. The results are summarised in Table III.

TABLE III.—*Comparison between the Behaviour of the $S^{35}$ Substance and Cytoplasmic Basophilia.*

| Treatment of cells. | $S^{35}$ activity. | Cytoplasmic basophilia. |
|---|---|---|
| 0·9 per cent NaCl 1 hr. at 37° C. . . . . | Not affected . | Removed. |
| 0·9 per cent NaCl 2 hr. at 37° C. . . . . | About ½ removed . | ,, |
| 10 per cent perchloric acid 2 hr. at 20° C. . | ,, ,, . | ,, |
| 1 N HCl 5′ at 60° C. . . . . . . | ,, ,, . | ,, |

Treatment with 0·9 per cent NaCl for 1 hour at 37° C. or for 2–3 hours at 20° C. removes most of the cytoplasmic basophilia as shown by subsequent staining with Giemsa, but it did not affect the $S^{35}$ content of the cells. 10 per cent perchloric acid for 2–3 hours at 20° C. also removes practically all cytoplasmic basophilia, but resulted in only a partial removal of the isotope. Hydrolysis with 1 N HCl for 5 minutes at 60° C. removes all cytoplasmic basophilia—but resulted also in only a partial removal of the isotope. Again it was observed that the eosinophil cells retained their original activity more than the neutrophil myelocytes.

It was not possible, therefore, to establish any close relation of the $S^{35}$ substance to the ribose nucleic acids of the cytoplasm. However, further experiments with specific enzymes (ribonuclease) are needed for confirmation.

c. *The uptake of methionine $S^{35}$.*

At the beginning of the experiments it was considered to be unlikely that $S^{35}$ administered as sulphate to the culture medium would be transformed by the cells to SH groups. An enzyme system in liver, however, which can incorporate limited amounts of sulphide sulphur into cystine has been reported (Smythe and Halliday, 1942). To exclude the possibility of the administered sulphate $S^{35}$ being present in the SH groups of proteins, it was decided to investigate the uptake of an $S^{35}$ labelled SH group; in a series of 10 experiments 15–25 $\mu$c./ml. dl-methionine $S^{35}$ was iucorporated into the culture medium. The summary of the findings is presented in Table IV. Methionine $S^{35}$ uptake was not limited to the

TABLE IV.—*Comparison between the Properties of $S^{35}$ Sulphate Uptake and Methionine $S^{35}$ Uptake.*

| | $S^{35}$ sulphate. | Methionine $S^{35}$. |
|---|---|---|
| Uptake | By myeloid cells only | By all types of bone marrow cells. |
| Localisation | Cytoplasm | Nucleus and cytoplasm. |
| Barbiturate | Inhibits uptake | Does not affect uptake. |
| 0·1 M $Ba(OH)_2$ or 0·1 M $BaCl_2$ | Removes all $S^{35}$ from cells | Does not remove $S^{35}$ from cells. |
| N HCl | Ditto | Removes about $\frac{1}{2}$ of $S^{35}$ from cells. |

myeloid series of cells as was $S^{35}$ sulphate; nucleated red cells also showed a marked uptake (Fig. 4, 5), as have lymphocytes from the peripheral blood of patients with lymphocytic leukaemia. Barbiturate did not inhibit methionine $S^{35}$ uptake as it did the $S^{35}$ sulphate uptake. The isotope was not removed from the smears of the methionine $S^{35}$ cultures by barium salts; and, finally, N HCl hydrolysis for 15 minutes at 20° C. resulted in only a partial removal of the methionine $S^{35}$ activity in contrast to complete removal of the $S^{35}$ sulphate activity.

It was concluded therefore that the $S^{35}$ substance is not present as protein sulphur in the cells.

### DISCUSSION.

These experiments show that mammalian cells other than cartilage are capable of utilising sulphate sulphur, and that in human bone marrow this ability is a specific function of the myeloid series of cells. The sulphate utilisation of bean root cells as observed by Howard and Pelc (1951) is an entirely different function. In the bean root cells the sulphate sulphur, in some form, is incorporated into the nuclei and is probably associated with DNA protein. In human bone marrow cells the localisation of the isotope is almost exclusively cytoplasmic, as far as can be ascertained by high resolution autoradiography.

$S^{35}$ sulphate administered *in vivo* to rats has been shown to be taken up into the chondroitin sulphate of the growing cartilage (Dziewiatkowsky, Benesch and Benesch, 1949; Dziewiatkowsky, 1949; 1951*a*, 1951*b*, 1952). Irrespective of whether sulphate or sulphite $S^{35}$ compounds were given to rats *in vivo*, activity was found in the bone marrow by Singher and Marinelli (1945). This finding has been confirmed by Dziewiatkowsky (1949, 1952) using whole bone autoradiographs and it was thought not to be chondroitin sulphate, but to be inorganic sulphate sulphur which is precipitated by $Ba(OH)_2$. This sulphur after the first 30 minutes of administration, is incorporated into a more complex compound from which the sulphur is slowly removed.

The $S^{35}$ substance in the myeloid cells of the bone marrow cultured *in vitro* is removed by barium salts. It is possible that the dose of $S^{35}$ sulphate given to the rats by Dziewiatkowsky was too small to be detectable in whole bone autoradiographs in the acid formalin fixed bones (5 $\mu$c./7-day-old rat in one series and 1·25 $\mu$c./7-day-old-rat in the other series of experiments).

At this stage of the work it is probably too speculative to attempt to correlate the specific $S^{35}$ sulphate uptake of the myeloid cells with the specific action of Myleran (1–4 dimethanesulphonyloxybutane) on the myeloid cells. The cytological effects of Myleran on myelocytes of the rat suggest a cytoplasmic mechanism (Koller and Timmis, 1953, personal communication).

### SUMMARY.

Cell suspensions of human bone marrows cultured *in vitro* show an uptake of $S^{35}$ sulphate from the culture medium. The $S^{35}$ sulphate uptake is limited to the myeloid series of cells; the lymphocytic and erythropoietic elements show no $S^{35}$ uptake.

The isotope is localised in the cytoplasm of the myeloid cells in the form of an organic complex removable by barium salts. There is no evidence that it is chondroitin or mucoitin sulphate, although it is probably related to such structures. It is not in the SH groups of proteins. The observations suggest a hitherto unknown specific function of the myeloid series of cells.

This work is part of an investigation carried out under a full grant from the British Empire Cancer Campaign.

### REFERENCES.

Bray, H. G., Gregory, J. E., and Stacey, M.—(1944) *Biochem. J.*, **38**, 142.

Doniach, L., and Pelc., S. R.—(1950) *Brit. J. Radiol.*, **23**, 184.

Dziewiatkowsky, D. D.—(1949) *J. biol. Chem.*, **178**, 197.—(1951*a*) *ibid.*, **189**, 187.—(1951) *J. exp. Med.*, **93**, 451.—(1952) *ibid.*, **95**, 489.

*Idem*, Benesch, R. E., and Benesch, R.—(1949) *J. biol. Chem.*, **178**, 931.

Howard, A., and Pelc, S. R.—(1951) 'Isotopes in Biochem.' London (Churchill.)

Lajtha, L. G.—(1952*a*) *J. clin. Path.*, **5**, 61.—(1952*b*) *Exp. Cell. Res.*, **3**, 696.

Rubin, P. S., and Howard, J. E.—(1950) Conf. Metab. Interrelat. Josiah Macy Jr. Found. N.Y., **2**, 155.

Singher, H. O., and Marinelli, L. D.—(1945) *Science*, **101**, 414.

Smythe, C. V., and Halliday, D.—(1942) *Fed. Proc.*, **1**, 134.

Sylven, B.—(1941) *Acta chir. scand.*, Suppl. 66.—(1945) *Acta Radiol.*, Suppl. 59.